# Solvent-Free Formation of Cyclodextrin-Based Pseudopolyrotaxanes of Polyethylene Glycol: Kinetic and Structural Aspects

**DOI:** 10.3390/ijms23020685

**Published:** 2022-01-08

**Authors:** Nerea Guembe-Michel, Adrián Durán, Rafael Sirera, Gustavo González-Gaitano

**Affiliations:** Department of Chemistry, School of Science, University of Navarra, 31080 Pamplona, Spain; nguembe.1@alumni.unav.es (N.G.-M.); adrianduran@unav.es (A.D.); rsirera@unav.es (R.S.)

**Keywords:** cyclodextrin, polyethylene glycol, polyrotaxane, kinetics, attenuated total reflectance, X-ray diffraction, derivative thermogravimetry

## Abstract

Pseudopolyrotaxanes (PPRs) are supramolecular structures consisting of macrocycles able to thread on a linear polymer chain in a reversible, non-covalent way, often referred to in the literature as “molecular necklaces”. While the synthesis and reaction mechanisms of these structures in solution have been widely described, their solvent-free production has received little attention, despite the advantages that this route may offer. We propose in this work a kinetic mechanism that describes the PPR formation in the solid phase as a process occurring in two consecutive stages. This mechanism has been used to investigate the spontaneous formation of a PPR that occurs when grinding α-Cyclodextrin (α-CD) with polyethylene glycol (PEG). In the threading stage, the inclusion of the polymer and subsequent release of the water molecules lodged in the cavity of the macrocycle cause vibrational changes that are reflected in the time-dependence of the FTIR-ATR spectra, while the further assembly of PPRs to form crystals produces characteristic reflections in the XRD patterns, due to the channel-like arrangement of CDs, that can be used to track the formation of the adduct in crystalline form. The effects that working variables have on the kinetics of the reaction, such as temperature, feed ratio, molar mass of the polymer and the introduction of an amorphous block in the polymer structure, have been investigated. The rate constants of the threading step increase with the temperature and the activation energy of the process increases at lower proportions of CD to PEG. This is attributed to the lower degree of covering of the polymer chain with CDs that reduces the hydrogen-bonding driven stabilization between adjacent macrocycles. The formation of crystalline PPR, which takes place slowly at room temperature, is markedly promoted at higher temperatures, with lower proportions of CD favoring both the formation and the growth of the crystals. The molar mass of the polymer does not modify the typical channel-like arrangement of packed PPRs but the conversion into crystalline PPR diminishes when using PEG1000 instead of PEG400. At a microscopic level, the crystals arrange into lamellar structures, in the order of hundreds of nm, embedded in an amorphous-like matrix. The introduction of a polypropylene oxide block in the structure of the polymer (Pluronic L62) renders poorer yields and a considerable loss of crystallinity of the product of the reaction. The methodology here proposed can be applied to the general case of inclusion complexes of CDs with drugs in the solid phase, or to multicomponent systems that contain polymers as excipients in pharmaceutical formulations along with CDs.

## 1. Introduction

Polyrotaxanes (PRs) are supramolecular structures consisting of a number of macrocycles non-covalently threaded on a linear polymer chain [1]. In a PR, the threaded rings cannot escape due to the presence of end-capping bulky groups covalently attached at the extremes of the axis polymer, hence the name of “molecular necklaces”. The absence of these stoppers results in a pseudopolyrotaxane (PPR), in which the macrocycles can enter and exit the polymeric chain, according to the chemical laws of equilibrium and kinetics [2,3]. These supramolecular constructs are gaining interest in different fields, mainly in drug delivery, since they can act as reservoirs of macrocycles that can be released either on-demand (by chemical or photochemical triggering) or during the slow degradation of the superstructure [4,5,6].

Most of the PRs and PPRs are built from cyclodextrins (CDs), cyclic oligosaccharides consisting of several glucopyranose units bound by α-1,4-glycosidic bonds forming a torus-like structure [7,8,9,10,11,12,13,14,15,16,17,18]. The three native CDs (α-, β- and γ-CDs, containing 6, 7 and 8 glucose residues, respectively) present very defined cavity sizes, which is the factor that mainly determines the affinity of the CD for a specific polymer. The threaded CDs form hydrogen bonds between adjacent macrocycles in different arrangements [7], producing a strong non-covalent network that stabilizes the supramolecular structure [7,11,12,13,14]. The formation of a CD-based PPR was first reported by Harada et al., [13] by precipitation from aqueous solutions of α-CD and a short polyethyleneglycol, PEG, in a 2:1 molar ratio ethylene oxide (EO) to α-CD [13,19,20]. Further investigations revealed the selectivity of the binding with the type of CD and monomer [1,13,21]. Thus, α-CD does not complex propyleneglycol, PPG, which has a wider cross-section than PEG. On the contrary, β- and γ-CD form more stable complexes with PPG than with PEG [2,9,11,13,21]. The threading mechanisms of CDs with different polymers to produce PPRs and PRs have been widely described in the literature [3,4,12,22], as well as their synthesis, characterization and applications [5,6,23,24,25,26].

While the vast majority of studies on these supramolecular structures deal with their synthesis in solution [1,2,3,4,7,9,10,11,12,13,14,20,21,22,24], the number of investigations in solid-state is much more limited [19,27,28,29,30,31,32]. Some studies have focused on the optimization of the synthesis and the characterization of the structures formed. For example, Liu et al. reported an effective way of synthesizing PPRs without solvents by grinding [29,30], and Kihara et al. described the end-capping of a PPR formed in solid-state by the aforementioned method for its further isolation and characterization [27]. Harada et al. found that tubular inclusion compounds could be formed in solid-state when mixing CDs with PEGs of different lengths [28]. The X-ray diffraction (XRD) patterns of the reaction products in both aqueous solution and solid-state showed similar structural features, despite the reaction conditions. Likewise, Peet et al. reported, for the reaction between α-CD and low-molecular-weight PEG, that the characteristic reflections in XRD, corresponding to the channel-like structure of the PPR, increased markedly with the temperature, and deduced the rate constants of the reaction, using a first-order kinetic model [19].

In general, the reaction rates of the formation of PRs and PPRs in solid form are slower than in solution. However, this limitation can be outweighed by the elimination of purification steps, once the product has formed, and by a cleaner, environment-friendly synthesis [29]. It is within this framework that we have investigated the formation of the PPR that forms spontaneously when mixing α-CDs and PEG. The combined use of time-dependent Fourier Transform Infrared Spectroscopy (FTIR) and XRD has permitted us to shed light on the mechanism of the reaction in the condensed phase, described as a sequential kinetic process in which the threading of the guest takes place first, followed by the packing of the adducts to produce the PPR in crystal form. The effects on the reaction yield and rates of temperature, proportion of the reactants in the feed, molar mass of the polymer and inclusion of amorphous domains in the polymer structure have been also analyzed. Derivative thermogravimetry (DTG) has been employed to determine the thermal features of the products formed, while the morphology at the microscale of the crystalline PPRs has been explored by Scanning Electron Microscopy (SEM).

## 2. Results and Discussion

### 2.1. Mechanism of the PPR Formation

In the literature, the formation of the PPR in the solid phase has usually been treated as a single-step process, described in terms of the changes occurring in the crystalline structure of the CD, which passes from the cage-structure to a channel-like structure [19]. In the scheme here proposed, the global reaction is split into two consecutive stages that can be analyzed independently: complex formation and ordered packing of the PPRs, each one with its corresponding rate constant, i.e.,:

$$\alpha CD+PEG\;\overset{k_{th}}{\to }\;PPR_{th}\;\overset{k_{c}}{\to }\;\;PPR_{c}$$

where *k_th_* and *k_c_* are the rate constants for the threading step and PPR packing (crystal formation), respectively. The reaction starts when the α-CD, in powder form and containing a certain amount of water molecules, is ground with the liquid polymer. The CD disperses in the PEG, which involves the rupture of the crystalline structure of the macrocycle and the release of the non-inclusion water of the CD. This process is controlled, among other factors, by the degree of diffusion of the reactants in the medium. Then, the free CDs thread the polymer to form the adduct, with the subsequent expelling of the water molecules contained in the cavities. Depending on the number of CDs available (feed ratio), several macrocycles can thread the polymer, with adjacent CDs interacting by hydrogen bonding between the hydroxyls at their rims. Finally, the partially or fully CD-covered PPRs pack to form crystals. The latter process can be monitored by XRD diffraction, while FTIR will reflect changes that occur in the vibrations of the reacting molecules along the whole process.

#### 2.1.1. Threading Step (Formation of the PPR)

**Figure 1:**
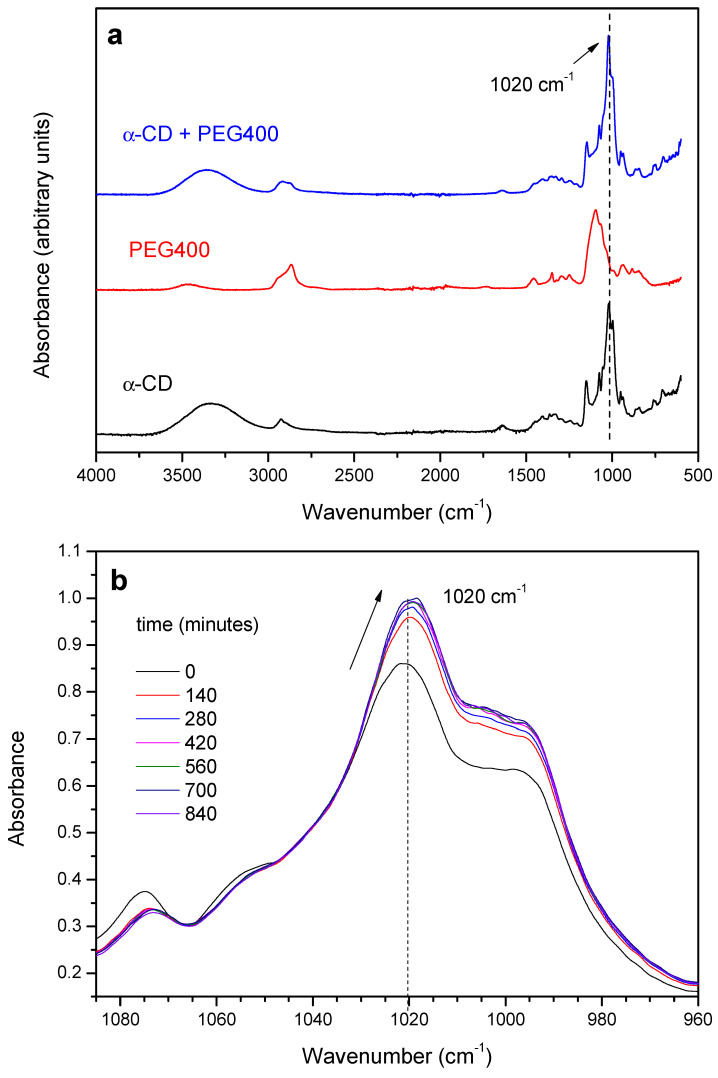
(**a**) FTIR-ATR spectra of α-CD, PEG400 and the reaction product after 120 min at 65 °C (1:1 feed ratio); (**b**) Expansion of the 960–1100 cm^−1^ region of the spectra an evolution with time.

The threading of the α-CD on the different PEGs has been monitored from the changes in the absorption of selected bands in the FTIR-ATR spectra over time. As an example, the vibrational spectra of the product of the reaction with PEG400 (red trace) at 65 °C (1:1 molar ratio) after 120 min is shown in Figure 1a (blue trace). The characteristic bands corresponding to the symmetric and antisymmetric O-H stretching vibrations, ν_s_(O-H) and ν_a_(O-H), of the α-CD appear in the interval 3000–3500 cm^−1^, and are present in both the non-reacted CD (Figure 1a, black trace) and the inclusion complex [33,34]. Since the CD contains a substantial amount of both hydration and inclusion water, changes in the areas/intensities of the 3000–3500 cm^−1^ band due to dehydration over time are expected, especially at the highest temperatures used, which will occur concomitantly with those resulting from the complex formation. The bands in the region 2800–3000 cm^−1^ are ascribed to C-H stretching modes, which are present in all the species (α-CD, PEG400 and the inclusion complex, Figure 1a from bottom to top, respectively) [33,34,35,36]. At lower energies, the HOH scissoring bending mode, δ(HOH), appears at 1640 cm^−1^ in both CD and the mixture, [35,36] as well as the OH bending around 1400 cm^−1^ (Figure 1a) [33]. Finally, the bands around 1150 cm^−1^ and 1020 cm^−1^ for both inclusion complex and CD are attributed to C-H bending, δ(C-H), and C-O stretching, ν_s_(C-O), modes, respectively [36]. In the case of PEG400, the bands that appear between 1100–1400 cm^−1^ are due to C-H bending modes δ(C-H), while the ν_s_(C-O) appears around 1080 cm^−1^.

**Figure 2:**
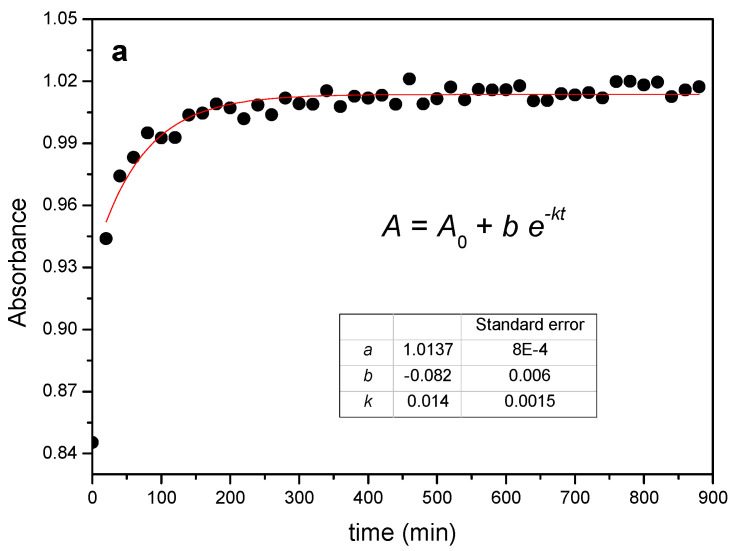

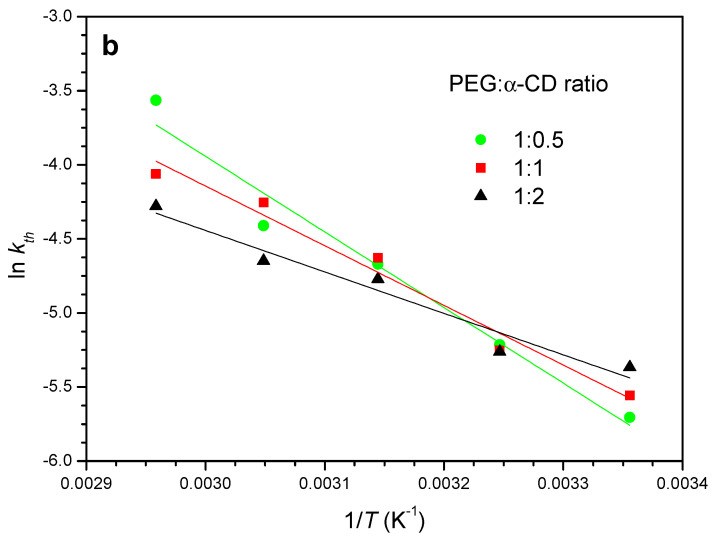
(**a**) Kinetic profile obtained from FTIR data for the reaction between PEG400 and α-CD (1:1 feed ratio) at 55 °C and corresponding fit. (**b**) Arrhenius plots for the reaction at different feeds.

The reaction between the α-CD and PEG results in the gradual increase of the absorbance of the C-O stretching mode of the macrocycle at 1020 cm^−1^ and in a slight red-shift of ca. 3 cm^−1^, with the occurrence of an isosbestic point at ca. 1040 cm^−1^, as shown in Figure 1b. The relatively small changes observed are in line with the formation of a non-covalent structure stabilized by van der Waals forces, in which the EO monomers replace the water molecules included in the CD. The evolution of the absorbance of the 1020 cm^−1^ band of the α-CD has been tracked as a function of time and temperature. As an example, Figure 2a shows the kinetic profile of the 1:1 mixture at 55 °C. The experimental data have been fitted to an exponential decay function with offset (see Supplementary Materials—Numerical analysis of FTIR and XRD data), from which the rate constant of threading, *k_th_*, can be obtained (Table 1).

The rate constants increase with the temperature in all cases and the reaction proceeds on average nearly four times faster when passing from room temperature to 65 °C, with a clear dependence on the reaction feed ratio. For example, at 25 °C the 1:0.5 ratio yields the lowest constant, while the trend inverts at 65 °C. This is a consequence of the different activation energies, *E_a_*, which can be obtained from the temperature dependence of the rate constants in the corresponding Arrhenius plots (Figure 2b).

Although the slopes in the plot do not differ much from each other, the differences in the calculated activation energies with the PEG:CD ratio are statistically significant (Table 1) and indicate that the energy barrier for the reaction becomes higher the lower the amount of CD is. The physical step that involves the dissolution and diffusion of the CD in the PEG must not account for this fact, since a less thick medium (lower CD proportion) would favor, in principle, the reaction. A plausible explanation for the observed trend in *E_a_* may be the different degrees of covering of the polymeric chain with α-CD at a given feed. According to the molecular dimensions of EO and CD, a single cavity could lodge two monomers, which implies that the saturation of the chain is reached with 4–5 macrocycles (9 EO for PEG400, on average) if the reaction takes place to the fullest extent. The formation of strong hydrogen bonds between adjacent CDs is a driving force that pulls the PPR formation, a factor that may be considerably reduced if the number of available CDs is small.

#### 2.1.2. Crystallization Step (Formation of Crystalline PPR)

**Figure 3:**
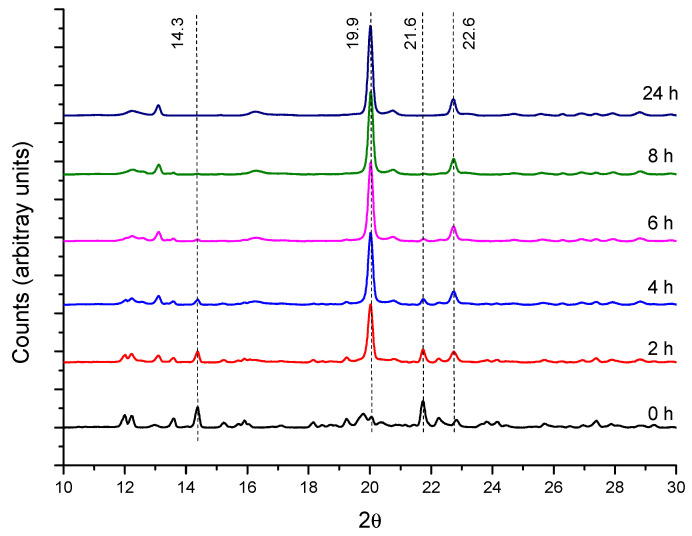
XRD patterns for PEG400:α-CD reaction (1:0.5 feed) at 50 °C as a function of time.

In CD-based PPRs, the arrangement of the macrocycles along the axis polymer and further packing produces channel-like structures with characteristic reflections in the powder XRD patterns, which are markedly different from the crystalline structure of the CD [14,37]. This is the case of the reaction of α-CD with PEG400 (Figure 3). The X-ray diffractograms show the reduction of the intensities of the reflections of the CD (2θ = 14.3 and 21.6°, cage-type structure), while those of the crystalline PPR (19.9 and 22.6°) increase. This same behavior is observed with 1:1 and 1:2 feed ratios (Figures S1 and S2).

**Figure 4:**
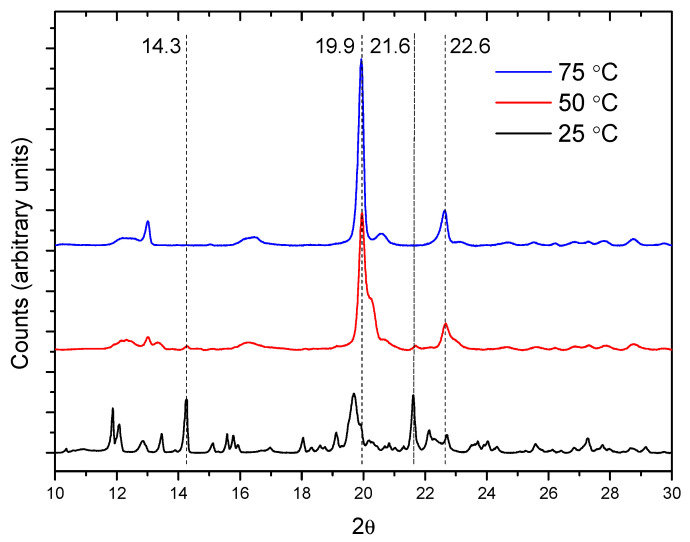
XRD patterns for the reaction between PEG400 and α-CD (1:1 molar ratio) after 24 h.

The temperature effect in the crystal formation is shown in Figure 4, which contains the diffractograms for the 1:1 mixture after 24 h. The 19.9° and 22.6° peaks of the PPR are more intense in the reactions at 50 and 75 °C, while the 14.3° and 21.6° ones (α-CD) are the weakest at these temperatures. At 25 °C, only small changes in the intensity of the peaks can be observed at any molar ratio (Figure S3). The ratio between the intensities of the reflections at 19.9° (PPR) and 14.3° (α-CD) can give an idea of the relative progression of the formation of crystalline PPR and helps to better compare the effect of the temperature and reaction feed in this second step (Figure S4). Clearly, the temperature favors the crystal formation rate, an effect that is more accentuated at lower CD proportion.

**Figure 5:**
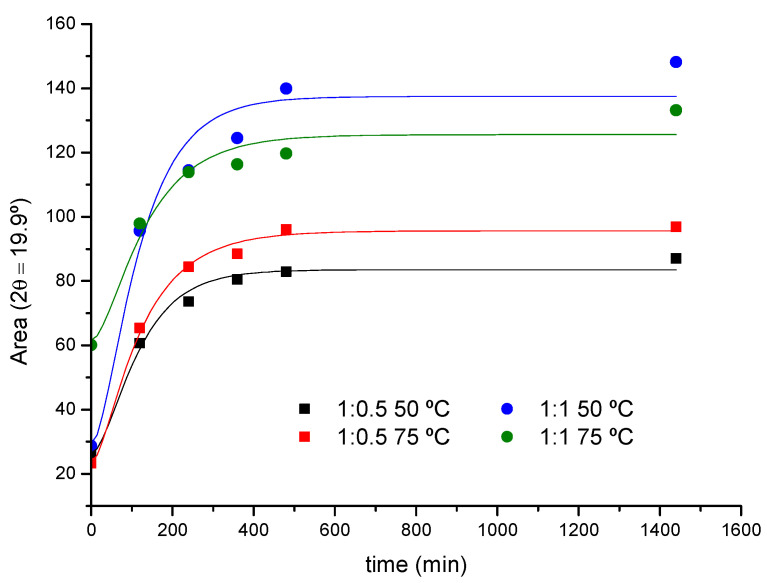
XRD kinetic profile for the reaction of PEG400 with α-CD (1:0.5 and 1:1 feeds, 50 and 75 °C).

The XRD data have been analyzed to evaluate the kinetic parameters of the crystal formation process, although in this case the number of points obtained per curve is considerably lower compared to FTIR due to practical reasons. As an example, Figure 5 shows the kinetic profile of the reaction of PEG400 (1:0.5 and 1:1 ratios) at 50 and 75 °C, obtained from the peak areas at 2θ = 19.9° (PPR). It can be seen how the crystalline adduct, *PPR_c_*, is practically formed after 6 h.

According to the proposed kinetic scheme, the decay of the crystalline *CD* follows a first-order rate law:

(1) $$\left[\alpha CD\right]={\left[\alpha CD\right]}_{0}\;e^{-k_{th}t}$$

The concentrations are expressed in mass percentage, as the reaction occurs in the solid phase. The rate of formation of the intermediate (*PPR_th_*) is the difference between its rate of formation and consumption

(2) $$\frac{d\left[PPR_{th}\right]}{dt}=k_{th}\;\left[\alpha CD\right]-k_{c}\;\left[PPR_{th}\right]$$

These expressions can be combined and integrated to obtain the concentration of the intermediate, *PPR_th_*, as a function of time:

(3) $$\left[PPR_{th}\right]=\frac{k_{th}}{k_{c}-k_{th}}\;\left({\left[\alpha CD\right]}_{0}\right)\left(e^{-k_{th}t}-e^{-k_{c}t}\right)$$

Finally, since $\left[\alpha CD\right]+\left[PPR_{th}\right]+\left[PPR_{c}\right]={\left[\alpha CD\right]}_{0\;}$ at all stages of the reaction (mass balance), the concentration of crystalline PPR will be

(4) $$\left[PPR_{c}\right]=\left({\left[\alpha CD\right]}_{0}\right)\left(1+\frac{k_{th}\;e^{-k_{c}t}-k_{c}\;e^{-k_{th}t}}{k_{c}-k_{th}}\right)$$

The rate constants of crystallization, *k_c_*, have been calculated for the 1:0.5 and 1:1 reaction feeds at 50 and 75 °C (Table 2), conditions for which the changes in the diffracted intensity permitted a more robust numerical analysis. To this effect, the areas of the 19.9° peak have been fitted to a modified form of Equation (4), introducing as a fixed parameter the value of *k_th_* previously obtained from FTIR in each case (see Supplementary Materials for details).

**Figure 6:**
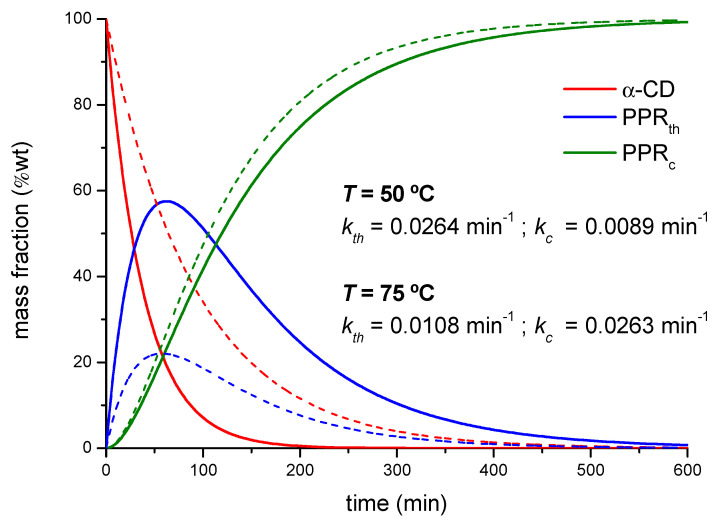
Calculated mass fractions of the different species in the reaction between PEG400 and α-CD, 1:1 reaction feed at 50 °C (dotted lines) and 75 °C (solid lines).

The *k_c_* values remain within the same order of magnitude as the threading constants but follow an opposite trend with the temperature. The threading process has been modeled as a first-order elementary reaction and, as such, the temperature increases the rate constant according to the Arrhenius law, as a difference to the subsequent packing of PPRs, which will be hampered by the thermal agitation of the system. The yield of the overall reaction in terms of *PPR_c_* formed will thus depend on the balance of both factors. According to the experimental rate constants obtained by XRD and FTIR, Equations (1)–(4) can be used to simulate the composition of all the species along the reaction (Figure 6). Under the experimental conditions used, the time of maximum concentration of intermediate (*PPR_th_*) is ca. 60 min in average for both temperatures and feed ratios.

At this point, it can be debated whether FTIR would be providing direct information on both processes (complex and crystal formation). This way, the time-dependence of the absorbance of the 1020 cm^−1^ band could be used to obtain simultaneously *k_th_* and *k_c_*, through Equation (4). If such were the case, the FTIR kinetic profile should match the scheme proposed (consecutive reaction occurring in two steps, each one following a first-order rate law, as described). However, irrespective of the dataset used (different temperatures and feed ratios), the fits produced always one rate constant close to the reported *k_th_* (Table 1) and the other tending toward infinity, as an indication of model over-parametrization, i.e., the mathematical description of the variation of the absorbance with time would only require one kinetic parameter. The fact that the FTIR spectra at 25 °C undergo changes similar to those at other temperatures while the XRD patterns are scarcely modified seems to confirm this point. Accordingly, the variations in the absorbance of the 1020 cm^−1^ band (Figure 1) must be ascribed solely to changes in the vibrations that take place when the CD threads the polymer, and not as a result of the subsequent packing of PPRs.

### 2.2. PPR Structure and Stability

**Figure 7:**
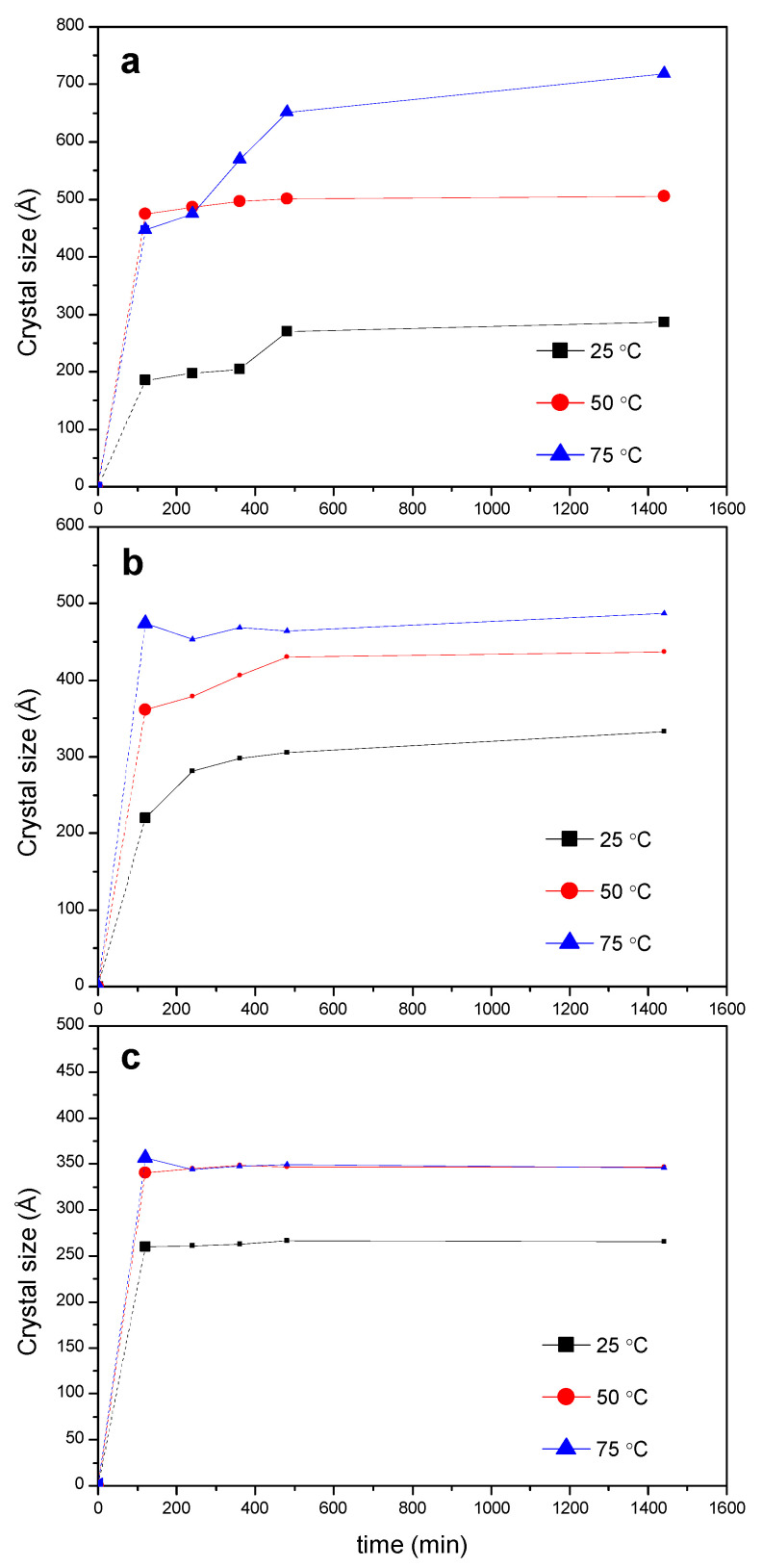
Evolution of the crystal size of the *PPR_c_* at different temperatures (calculated from the 2θ = 19.9° peak) for different reaction feeds PEG400:α-CD: (**a**) 1:0.5; (**b**) 1:1; (**c**) 1:2.

Relevant information on the evolution of crystal sizes overtime of the *PPR_c_* can be extracted from the Debye–Scherrer equation (Equation (1)), applied to the intense reflection at 2θ = 19.9°, as shown in Figure 7. The size of the crystals depends on temperature, time and feed ratio PEG:CD. A significant increment in crystal size occurs in the first hours of the kinetic run, reaching the highest value for the 1:0.5 mixture at 50 °C and 75 °C (Figure 7a). The temperature favors the growth of the crystals at any feed ratio (the little advance in the crystal formation at 25 °C is also reflected in the crystal size plots, the crystals showing the lowest sizes for a given PEG:CD ratio at such temperature). For the 1:2 feed (Figure 7c) the maximum size is reached at lower times, at any temperature, being the crystals the smallest, in agreement with the lowest conversions in *PPR_c_* observed (Figure S4). The lower rate constant of the threading step for this ratio (Table 1) may be in the origin of this behavior, to which contributes the effect of a thicker reaction medium that hampers the further PPR packing and, therefore, the crystal growth.

**Figure 8:**
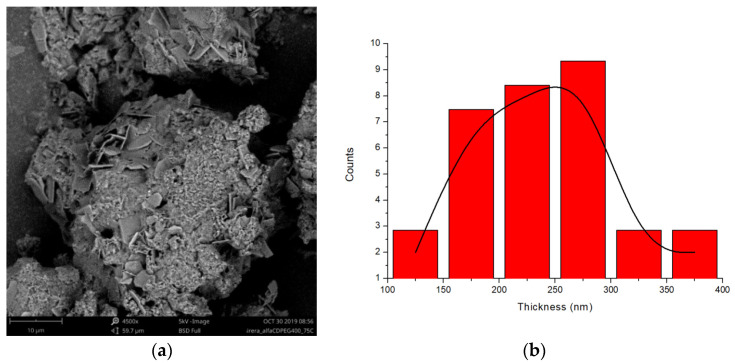
(**a**) SEM micrograph of the reaction of PEG400 and α-CD (1:2 molar ratio) at 75 °C (BSE electrons, 4500×, 5 kV); (**b**) Thickness distribution of the lamellae.

The microstructure of the *PPR_c_* has been explored by SEM. Figure 8a shows the SEM micrograph (BSE detector) obtained for PEG400 and α-CD (1:2 molar ratio) at 75 °C, in which the presence of lamellar structures embedded in an amorphous-like matrix, is observed, (see additional micrographs Figures S5 and S6). The thickness distribution of the lamellae (Figure 8b), calculated using selected regions of the micrograph, is broad and renders an average thickness of around two hundred nm. The formation from aqueous solution of lamellar microstructures of PPRs of CDs and PEG-containing copolymers has been described in the literature, with similar thicknesses [37] to those observed in solid form, although better defined and more extended, given the superior efficiency and homogeneity condition for the reactants mixing in solution.

**Figure 9:**
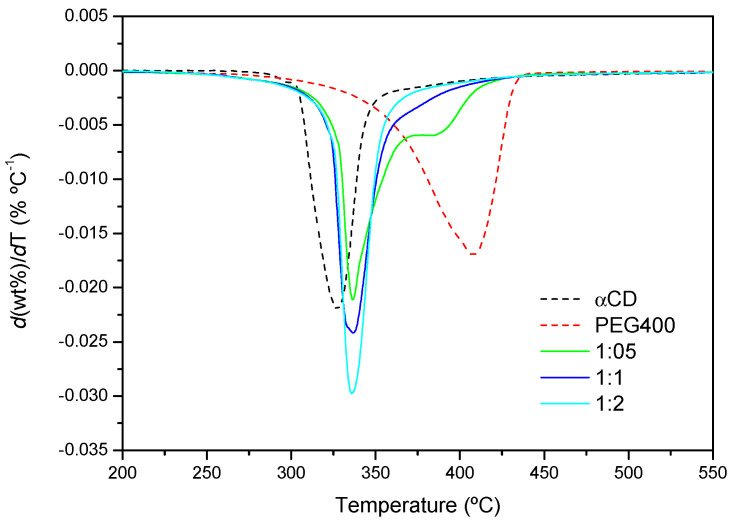
DTG curves of PEG400, α-CD and the products formed after the reaction for 24 h at 75 °C (1:0.5, 1:1 and 1:2 PEG400:α-CD molar ratios).

The thermal stability of the products after 24 h of reaction has been investigated by TGA. Figure 9 shows the first derivative of the normalized TGAs (DTG profiles) obtained from the reaction of PEG400 with α-CD at 75 °C at different reaction feeds. The maximum velocities of degradation of α-CD and PEG correspond to 326 °C and 409 °C, respectively. By contrast, the product of the reaction has its maximum decomposition speed at virtually the same temperature, ca. 378 °C, in-between the temperatures of the CD and the polymer, irrespectively of the temperature at which the reaction has been conducted. The similar decomposition patterns point, to a similar structure of the product at a molecular level in all cases, in line with the XRD findings. Additionally, for the 1:1 and 1:2 ratios, only a single thermal decomposition process is observed, in contrast to the 1:0.5 mixture, in which a second peak appears. This peak resembles that of the polymer alone, but its relative area, deduced from the deconvolution of the differential thermogram, is 28% lower than the actual weight percentage of the polymer at this molar ratio in the feed (40% PEG), and it appears at a considerably lower temperature than PEG (nearly 25 °C). These evidences point to thermal decomposition of polymer chains that are partially threaded by CDs, without reaching saturation, which is more likely to occur at low proportions of α-CD, rather than to non-reacted PEG. Regarding the pre-decomposition temperature range, from 20 °C to 200 °C, there is a small mass loss corresponding to water that has not been released during the formation of the inclusion complex (data not shown in the plots). As expected, this loss is lower the higher the temperature of the reaction is (for example, for the 1:0.5 feed, it is 1.8% at 50 °C, and 0.5% at 75 °C).

### 2.3. Effects of the Polymer Chain Length and Structure

**Figure 10:**
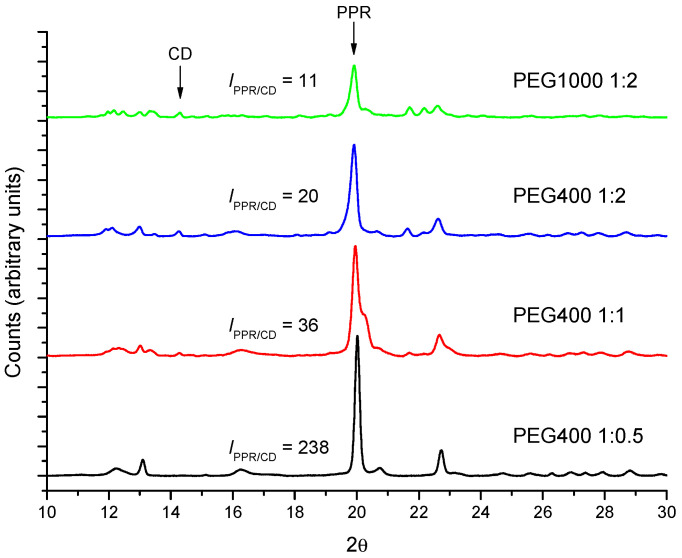
Comparison between the diffractograms of different molar ratios PEG400 and PEG1000 with α-CD after 24 h at 50 °C.

The kinetics of PPR formation using PEG1000 has been monitored by FTIR using the same procedure as for PEG400. As an example, Figure S7 shows the kinetic profile for the reaction between PEG1000 and α-CD (1:2 molar ratio) at 50 °C. The reaction proceeds more slowly (*k_th_* = 6.7 ± 0.6) × 10^−3^ min^−1^, than with shorter PEG400 (Table 1). On the other hand, the molar mass of the polymer does not seem to change the type of structure formed in the solid phase (the characteristic reflections in XRD are the same as in Figure 10) but it does affect the progression of the formation of the crystals, evaluated as the ratio of intensities 19.9° (PPR)/14.3° (CD). For a given polymer, the conversion into crystalline PPR increases the lowest the proportion of CD is, while it diminishes when increasing the molar mass of the PEG.

**Figure 11:**
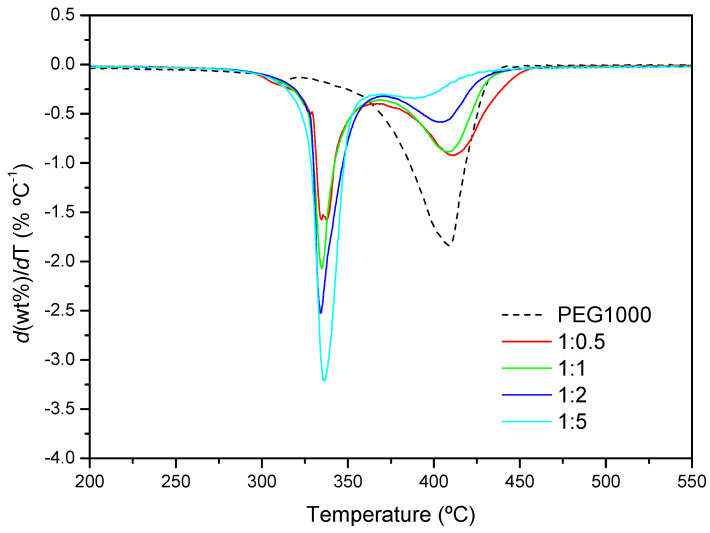
DTG profiles of PEG1000 and the product formed after 24 h at 50 °C (1:0.5, 1:1, 1:2 and 1:5 molar ratios).

Figure 11 shows the DTG curves for the PEG1000-αCD reaction after 24 h. The decomposition speed is maximum around 336 °C, irrespectively of the feed. As with PEG400, the nearly identical peak temperatures would confirm the similar structural features of the PPR with both polymers. By contrast, a second thermal process takes place in all cases, whose proportion diminishes with the increasing content in CD, while the temperature of maximum degradation shifts to lower values. The explanation provided above for PEG400 can be argued here, i.e., the lower the proportion of CD, the more likely is that segments of polymer remain uncovered with macrocycles, making the PPR more “PEG-like”.

Finally, we consider the effects of the introduction in the polymer structure of a block that does not crystallize on its own, like PPO. We have chosen Pluronic L62, a linear block copolymer with 30 units of PO forming a middle block, flanked by blocks of 5 EOs, in a molar ratio L62:α-CD 1:5, to achieve the saturation of the PEO blocks. Given the selectivity of the α-CD for the EO, the threading cannot proceed past the PPO moiety and thus the macrocycles will be confined at the PEO blocks.

**Figure 12:**
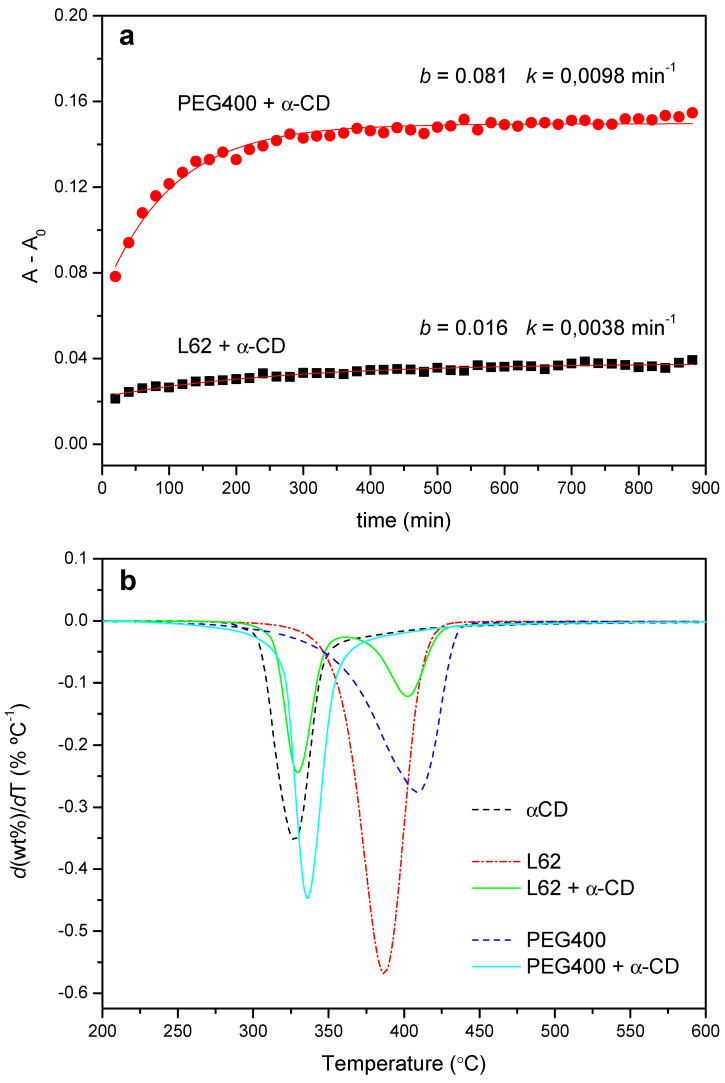
(**a**) Kinetic profiles for the reaction between L62 and α-CD (1:5, 50 °C) and PEG400 and α-CD (1:2, 55 °C). (**b**) DTG curves obtained from the PEG400 and L62 reactions with α-CD (1:2 and 1:5 feeds, respectively) after 24 h at 50 °C.

Figure 12a shows the FTIR kinetic profile of the reaction with L62 (1:5 molar ratio), with its corresponding curve fit. The reaction proceeds at a considerably lower rate than the one with PEG400, at the same ratio EO/CD and similar temperature (*k_th_* = 3.8 × 10^−3^ min^−1^, versus 9.6 × 10^−3^ min^−1^ for PEG400, Table 1). Likewise, the pre-exponential factor of the fit, which can be interpreted as a relative measure of the extension of the reaction (Figure 2), indicates that the formation of PPR is clearly less efficient (*b* = 0.016 for L62 versus 0.081 for PEG400).

The lower rate of the threading stage limits the further packing of the PPRs. This is evidenced in the XRD patterns, which practically match the diffractogram of the CD, even when the reaction time extends up to one week (data not shown), but also in the thermal behavior of the products. Figure 12b shows the normalized DTG trace after 24 h of reaction of PEG400 with α-CD at 50 °C together with that of the Pluronic. The copolymer decomposes in a broad range of temperatures, with a maximum speed of decomposition at 387 °C, while the product shows one process at 328 °C (practically the same as the CD alone, 327 °C), ascribed to the decomposition of the non-reacted CD plus a reduced amount of PPR, and another one at 402 °C, corresponding to the thermal decay of the PPG moiety of the copolymer. Therefore, the introduction of a PPO block in the polymer structure, albeit it does not fully impede the threading step, hinders the subsequent packing of the PPRs thus diminishing the yield of the crystallization step.

## 3. Materials and Methods

### 3.1. Chemicals

The native α-CD was obtained from Wacker (Munich, Germany), with purity ≥98% and 7.61% water content, as determined by thermogravimetry. PEG with 400 and 1000 g mol^−1^ average molar mass (PEG400 and PEG1000, respectively hereafter) were both purchased from Panreac (Barcelona, Spain), of analytical degree. Linear block copolymer Pluronic L62 (2500 g mol^−1^ molar mass, 30 propylene oxide (PO) units forming the middle block, flanked by blocks of 5 ethylene oxide (EO) monomers) was a gift from BASF (Ludwigshafen, Germany).

### 3.2. Preparation of the Pseudopolyrotaxanes

The PPRs were produced in the solid phase by hand-grinding the reactants in an agate mortar, following the procedure described by Peet et al. [19]. The sample texture passed over time from a wet slurry when mixing the reactants to a dry paste (typical grinding times were 5 min). Different molar ratios PEG:CD in the reaction feed were used (1:0.5, 1:1 and 1:2). Then, the sample was either kept in an oven at the target temperature for a fixed time (for further X-ray diffraction and thermogravimetric analysis) or readily transferred to the infrared spectrometer for FTIR kinetic analysis (see Supplementary Materials, Table S1, for details on the techniques used and experimental conditions).

### 3.3. FTIR-ATR Characterization and Kinetics

Infrared spectra were recorded in the range 600–4000 cm^−1^ on a Shimadzu (Kyoto, Japan) IRAffinity-1S spectrometer equipped with a thermostated Attenuated Total Reflectance (ATR) accessory (Golden-Gate, from Specac) with a diamond window. Each temperature run (ranging from 25 °C to 75 °C) consisted of 45-time steps every 20 min (total time of 24 h). Along the kinetic run, 32 scans were collected at each time step, using Happ-Genzel apodization to render the Fast Fourier Transform (FFT) of the averaged interferogram. In order to improve the statistics, the resolution of the resulting spectra was incremented from the experimental setup of 2 cm^−1^ to 0.125 cm^−1^ by polynomial interpolation with OMNIC 6.0 software [38]. Then, the maximum absorbance of the band at 1020 cm^−1^, assigned to the macrocycle C-O stretching, was used to build the kinetic curve, from which the rate constants were obtained via non-linear least-squares fitting using Origin Pro 8.5 software, [39] as explained in Supplementary Materials. The activation energy, *E_a_*, has been determined from the temperature dependence of the rate constant by using the Arrhenius equation, $k=A\;e^{-E_{a}/RT}$, where *k* is the rate constant; *A* is the pre-exponential factor; and *R* and *T* are the universal gas constant and the absolute temperature, respectively.

### 3.4. XRD Characterization

The kinetics of formation of crystalline PPR were analyzed at 25, 50 and 75 °C by X-ray powder diffraction in a Bruker (Billerica, MA, USA) D8 Advance diffractometer employing the Cu K_α1_ radiation (1.5406 Å), from 5° to 40° (2θ) each 0.01° at one second per step. For the experiments at 25 °C, PEG and CD were mixed and transferred readily to the diffractometer for the kinetic run, while at higher temperatures the sample was kept in the oven between measurements (typically every 2 h).

The XRD kinetic curves were plotted using the areas of the characteristic peak of the PPR (2θ = 19.9°), associated with the channel-like arrangement of threaded CDs along the polymer [9,14,19,37,40]. The Debye–Scherrer equation was then used to study the evolution of the average crystal sizes of the PPRs over time (Equation (5)), which relates the size of the growing crystallites to the width of the corresponding diffraction peak:

(5) $$D=\frac{K\;\lambda }{\beta \;\cos\theta }$$

where *D* is the average size of the microcrystals; *K* is the shape factor, which varies with the actual shape of the crystallite (set to 0.89); *λ* is the wavelength of the X-ray source; *β* is the width of the peak at 2θ = 19.9° at half of the maximum intensity, after subtracting the instrumental width; and θ is the Bragg angle. The calculations were performed with the algorithm implemented in the software of the diffractometer, DIFFRAC.EVA [41].

### 3.5. Derivative Thermogravimetric Analysis

Around 15 mg of the products formed after 24 h of reaction at the different temperatures were introduced in Al_2_O_3_ crucibles and the thermograms recorded in a TG-sDTA 851 Mettler-Toledo (Greifensee, Switzerland) thermal analyzer, using a heating ramp of 10 °C min^−1^, static air atmosphere and a temperature range from 25 to 700 °C. The derivative thermogravimetric (DTG) curves of the sample weight with the temperature and peak analysis were performed with Origin Pro 8.5 software [39].

### 3.6. Scanning Electron Microscopy

The microstructure of the products formed was explored by Scanning Electron Microscopy (SEM), using a Phenom ProX Desktop SEM (Thermo Fisher Scientific, Waltham, MA, USA). The samples were measured as received (without metal or carbon coating) and placed in a special sample-holder that largely removes the excess of negative charge caused by the electron beam, applying electron acceleration potentials from 5 to 15 kV. Backscattered electrons (BSE) or secondary electrons (SE) were used to obtain the micrographs. Numerical treatment of the images to obtain the thickness of the lamellae was accomplished with ImageJ software [42].

## 4. Conclusions

The spontaneous, solvent-free formation of PPRs that occurs when mixing α-CD and liquid PEG has been investigated by proposing a mechanism that considers that the reaction proceeds consecutively in two stages: formation of the supramolecular structure (*PPR_th_*), by diffusion of the CD in the liquid PEG and further threading on the polymer, and subsequently ordered packing of the adducts to produce the crystals (*PPR_c_*). The threading step, which entails changes in the vibrational spectra due to the entrance of the EO monomers in the CD cavity and the release of the included water molecules, has been studied by FTIR-ATR spectroscopy. The progression of the crystallization step is evidenced in the appearance of a distinct XRD pattern that evolves over time, in which the reflection at 2θ = 19.9°, the fingerprint of the channel-like structure of the molecular necklace, can be used to track the formation of crystalline PPR.

The rate of the threading step increases with the temperature, with kinetic constants that are within the same order of magnitude as the crystallization rate ones, although following an opposite trend. For PEG400:α-CD, the activation energy of threading depends on the feed ratio, increasing the lower the content in CD is. The different covering extension of the polymeric chain in each case is proposed as a key factor to account for this effect since a reduced number of threaded CDs hinders the formation of the hydrogen-bond network between adjacent CDs. This partial covering of the PEG chain at a low proportion of CD is manifested in the differential thermograms as an additional decomposition process that occurs at lower temperatures than the polymer alone, in all cases.

The formation of the crystalline adduct from PEG400 and PEG1000 with α-CD, which takes place slowly and with poor yield at room temperature, is improved at higher temperatures, as determined from the kinetic analysis of the XRD data, lower proportions of CD favoring both the progression of the *PPR_c_* formation and the growth of the crystals. The molar mass of the polymer does not modify the channel-like arrangement of packed PPR, although the relative conversion into crystalline PPR is lower with PEG1000 than with PEG400. At a microscopic level, the PPR crystals arrange into characteristic lamellar structures embedded in an amorphous-like matrix. On the other hand, when the reaction is carried out with Pluronic L62, in which the PEO blocks are separated by a middle PPO block, the threading of the macrocycle occurs at a much lesser rate and extent than with PEG of equivalent length, which limits the further formation of crystalline PPR.

From the methodological viewpoint, the mechanism proposed to investigate the formation of CD-based PPRs with the combined use of XRD and FTIR techniques, which provide complementary information on the different phenomena occurring along the reaction, has proven useful in understanding the kinetics of these reactions in the solid phase, as well as the effect of working variables on the structures formed. This scheme may provide a handle for the design of pharmaceutical formulations in the solid phase comprising CD complexes and polymers, in which competitive interactions between the different components are bound to occur.

## Figures and Tables

**Table 1:** Rate constants obtained from FTIR for the reaction of PEG400 with α-CD at different feeds (PEG:CD) and temperatures.

*T* (°C)	*k_th_* (min^−1^)		
	1:0.5	1:1	1:2
25	(3.3 ± 0.2) × 10^−3^	(3.9 ± 0.3) × 10^−3^	(4.7 ± 0.4) × 10^−3^
35	(5.4 ± 0.2) × 10^−3^	(5.2 ± 0.3) × 10^−3^	(5.2 ± 0.4) × 10^−3^
45	(9.4 ± 0.3) × 10^−3^	(9.8 ± 0.4) × 10^−3^	(8.5 ± 0.5) × 10^−3^
55	(12.1 ± 0.8) × 10^−3^	(14 ± 1) × 10^−3^	(9.6 ± 0.6) × 10^−3^
65	(28 ± 2) × 10^−3^	(17 ± 1) × 10^−3^	(13.9 ± 0.9) × 10^−3^
*E_a_* (kJ mol^−1^)	42 ± 4	34 ± 4	23 ± 3

**Table 2:** Rate constants of PPR crystal formation from XRD for the reaction of PEG400 with α-CD.

Molar Ratio	2θ	*k_c_* (min^−1^)	
		50 °C	75 °C
1:0.5	19.9°	(23 ± 7) × 10^−3^	(10 ± 2) × 10^−3^
1:1	19.9°	(26 ± 2) × 10^−3^	(9 ± 3) × 10^−3^

## Data Availability

The datasets generated and/or analyzed are available from the corresponding author upon request.

## Supplementary Materials

Supplementary materials can be found at www.mdpi.com/article/10.3390/ijms23020685/s1.
