# Supplementary material for: Solvent-Free Formation of Cyclodextrin-Based Pseudopolyrotaxanes of Polyethylene Glycol: Kinetic and Structural Aspects
Source: Int J Mol Sci. 2022 Jan 8;23(2):685. doi: 10.3390/ijms23020685 (PMC8775932; doi:10.3390/ijms23020685)
Supplement: Supplementary file 1 [file ijms-23-00685-s001.zip › ijms-1523491-supplementary.pdf]

*Supplementary Materials*

**Table S1.** Summary of experiments performed for each molar ratio, polymer type and temperature.

| Sample | Molar Ratio | Temperature (°C) | Technique(s) |
|---|---|---|---|
| PEG400 : $\alpha$-CD | 1:0.5 | 25, 50, 75 | TGA, XRD (kinetics) |
| | | 25, 35, 45, 55, 65 | FTIR (kinetics) |
| | 1:1 | 25, 50, 75 | TGA, XRD (kinetics) |
| | | 25, 35, 45, 55, 65 | FTIR (kinetics) |
| | 1:2 | 25, 50, 75 | TGA, XRD (kinetics) |
| | | 25, 35, 45, 55, 65 | FTIR (kinetics) |
| | | 75 | SEM |
| PEG1000 : $\alpha$-CD | 1:0.5 | 50 | TGA, XRD, FTIR |
| | 1:1 | 50 | TGA, XRD, FTIR |
| | 1:2 | 50 | TGA, XRD, FTIR (kinetics) |
| | 1:5 | 50 | TGA, XRD, FTIR |
| L62 : $\alpha$-CD | 1:5 | 50 | TGA, XRD, FTIR (kinetics) |

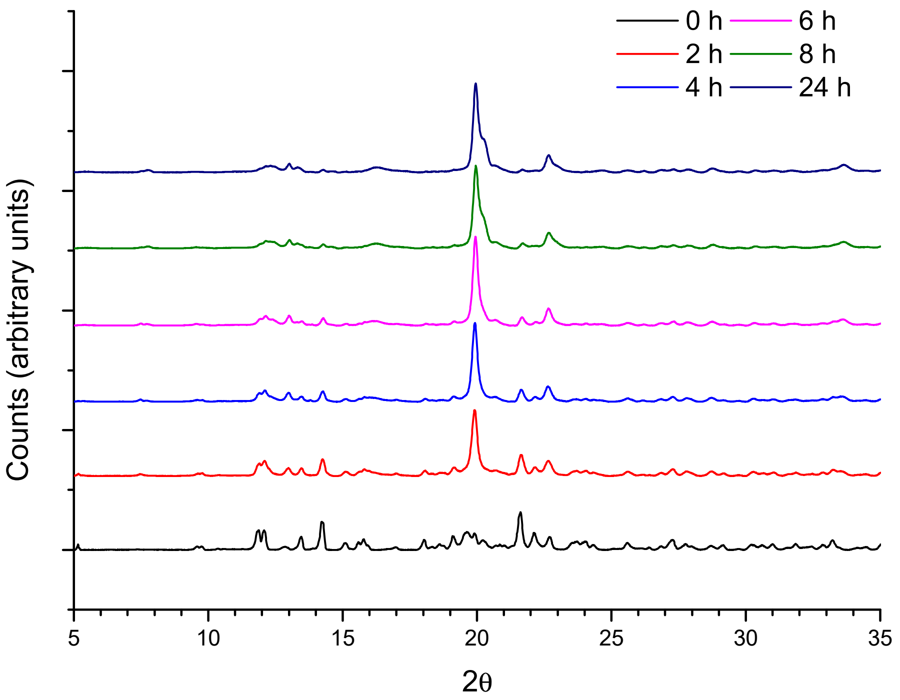


**Figure S1.** XRD patterns for the reaction PEG400 with $\alpha$-CD (1:1 feed) at 50 °C as a function of time.

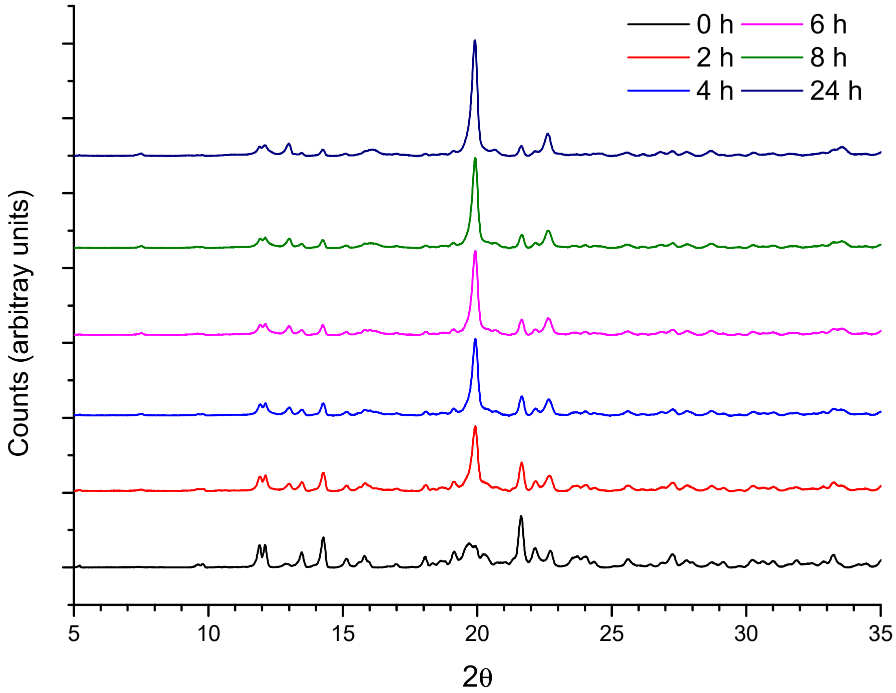


**Figure S2.** XRD patterns for the reaction PEG400 with $\alpha$-CD (1:2 feed) at 50 °C as a function of time.

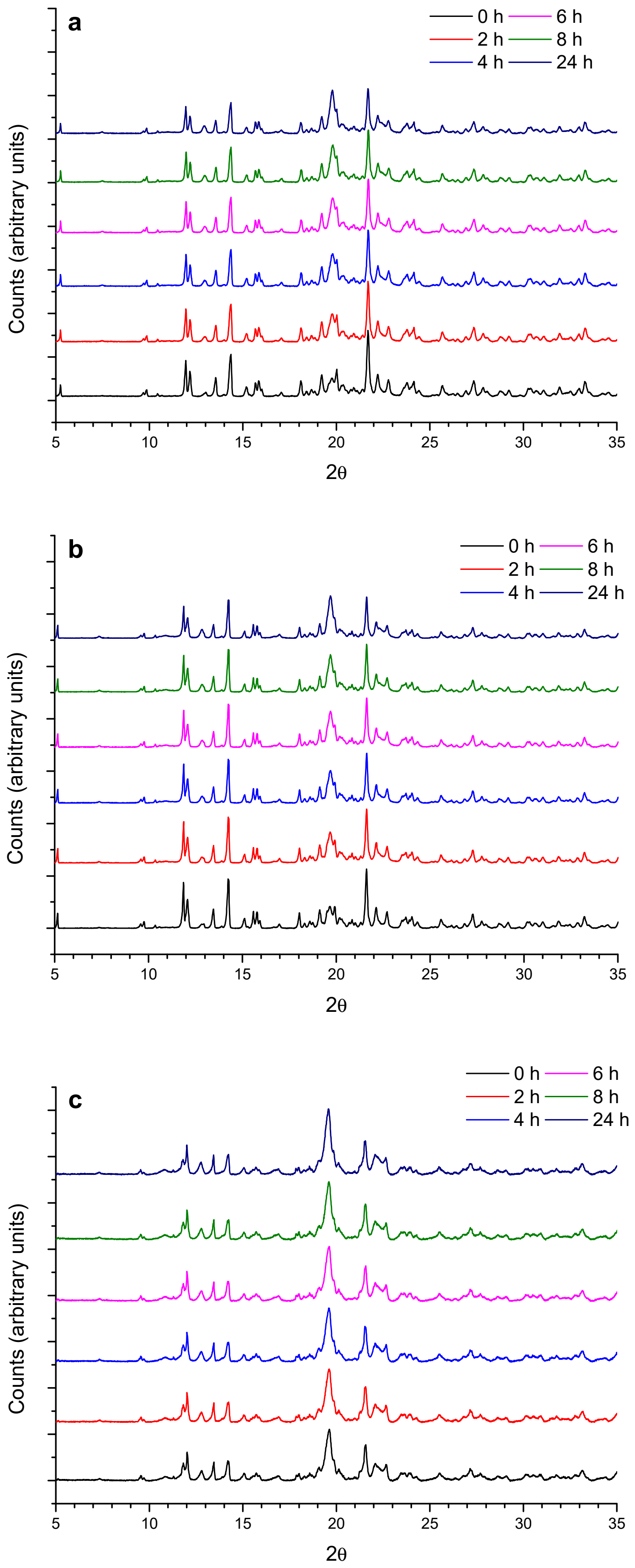


**Figure S3.** XRD patterns for the reaction PEG400 with $\alpha$-CD at 25 °C as a function of time. (**a**) 1:0.5, (**b**) 1:1, (**c**) 1:2 feeds.

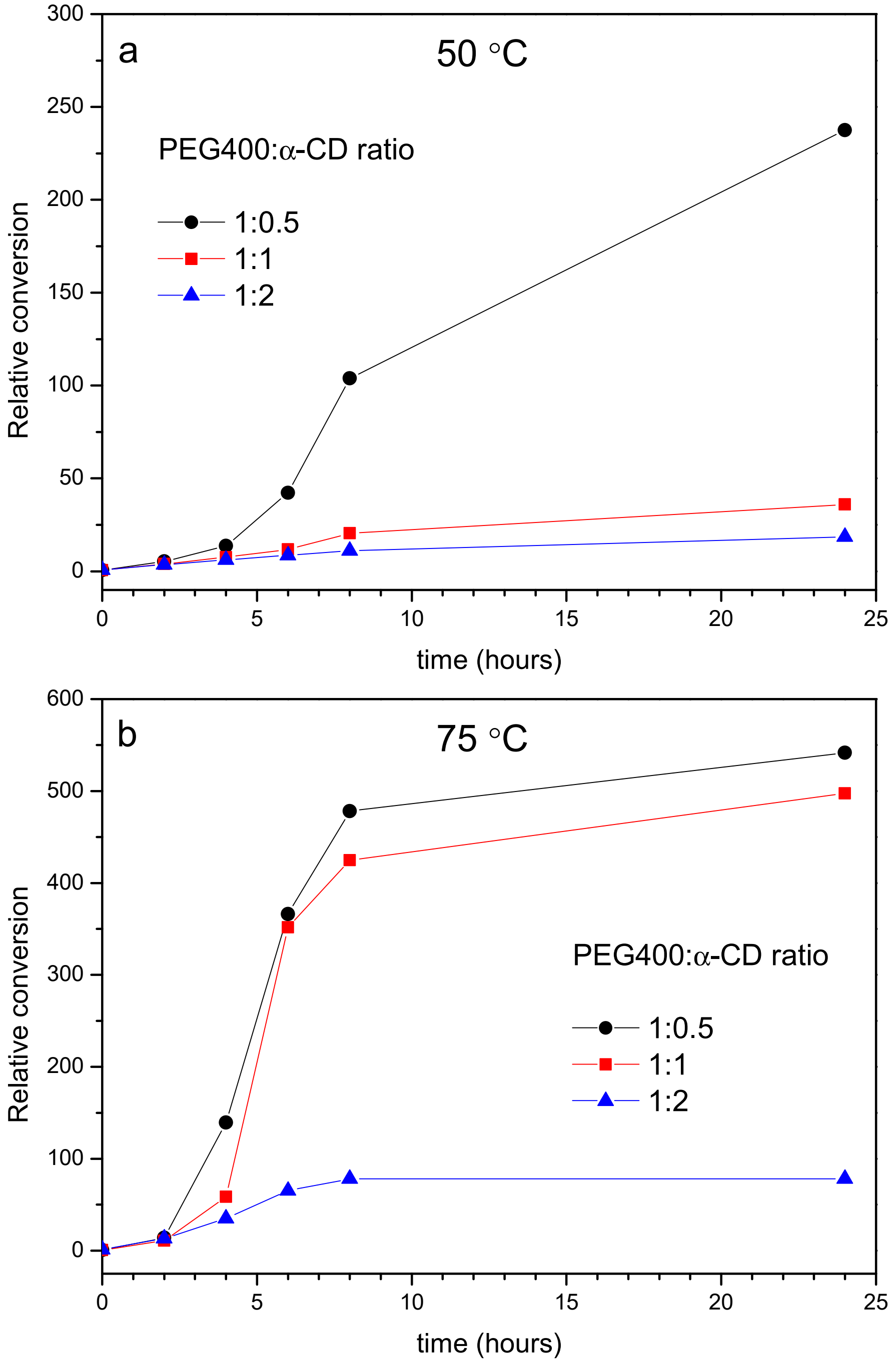


**Figure S4.** Evolution of the relative conversion, calculated as $I_{19.9°}/I_{14.3°}$, at different PEG400:α-CD feed ratios at: (**a**) 50 °C; (**b**) 75 °C.

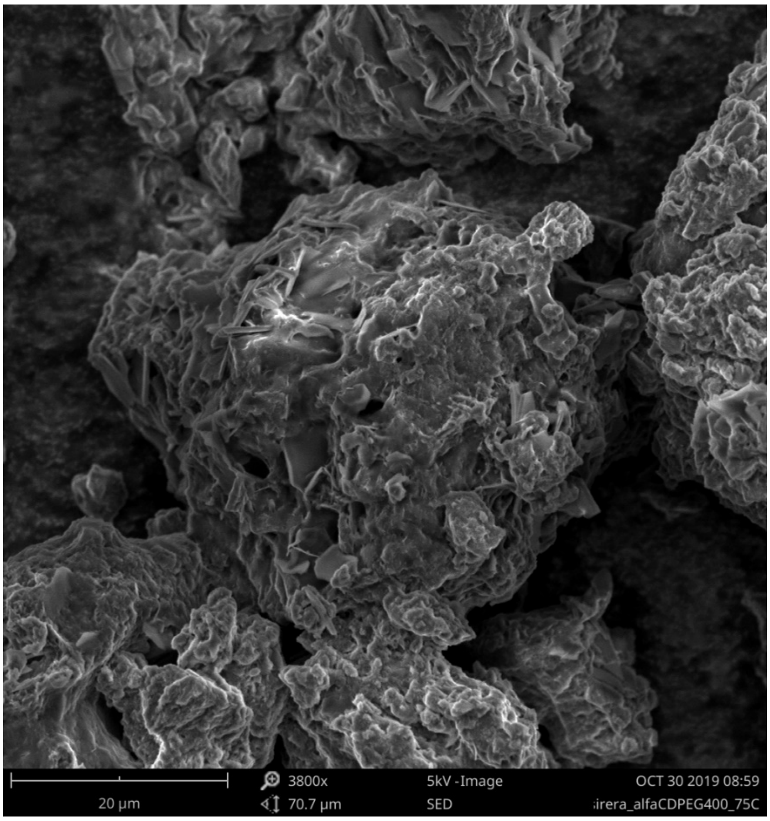


**Figure S5.** SEM micrograph of the reaction of PEG400 and $\alpha$-CD (1:2 molar ratio) at 75 °C (SE electrons, 3800× and 5 kV).

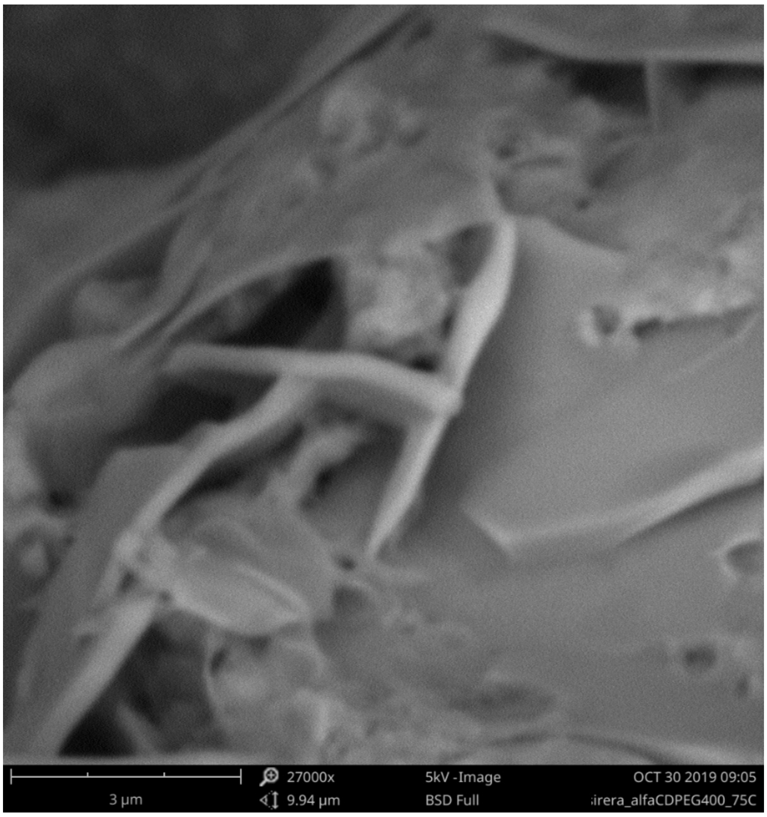


**Figure S6.** SEM micrograph of the reaction of PEG400 and $\alpha$-CD (1:2 molar ratio) at 75 °C (BSE electrons, 27,000× and 5 kV).

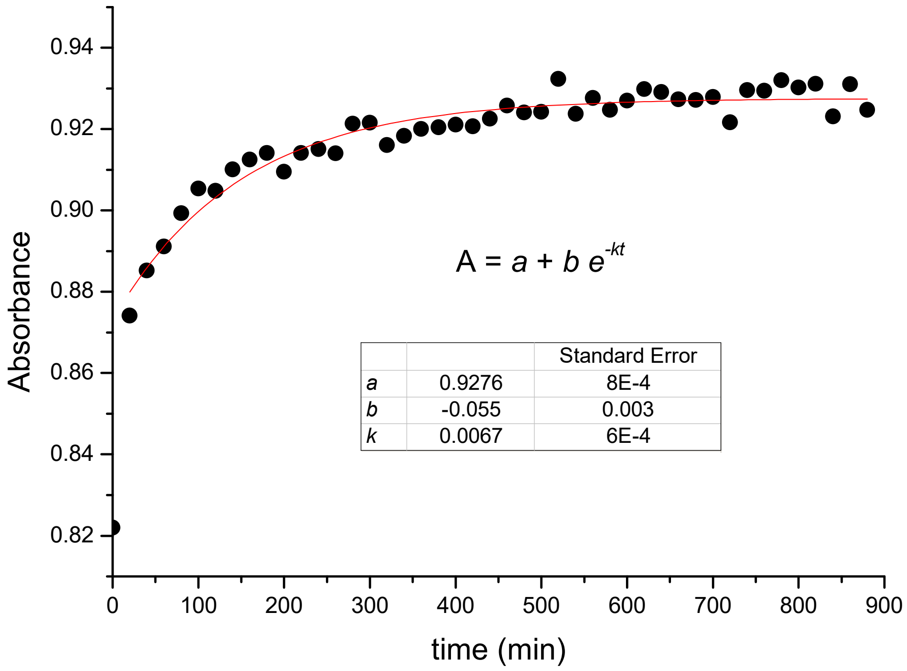


**Figure S7.** Kinetic profile obtained from FTIR data for the PEG1000 and $\alpha$-CD reaction (1:2 feed ratio) at 50 °C and corresponding fit.

## Numerical analysis of FTIR and XRD data

*a) FTIR data treatment*

The spectra collected at each time step were exported to OMNIC 6.0 software (Thermo Nicolet Corporation. Thermo Fisher Scientific, Madison, WI, USA) and the resolution enhanced from 2 $cm^{-1}$ to 0.125 $cm^{-1}$. The files were then transferred to OriginPro 8.5.0 software (OriginLab Corporation, Northampton, MA, USA) as a set of spreadsheets. Then, the absorbance at the maximum (1020 $cm^{-1}$) was extracted at each time (20 min up to 24 h) and the resulting curve fitted by non-linear least squares to the function:

$$A = A_0 + b\, e^{-kt}$$

Where $A_0$ represents the offset level, $b$ is a pre-exponential factor and $k$ is the rate constant of the threading step, $k_{th}$. Kinetic data were fitted with the implemented non-linear least squares modulus in OriginPro, using the default Levenberg-Marquardt algorithm. The first point of each curve (zero time, corresponding to the mixture of the reactants) is affected by the largest error and has not been considered into the fits.

*b) XRD data treatment*

The reflection at 2θ = 19.9°, associated to the $PPR_c$, was integrated at each time and the resulting curves area vs. time (Figure 5) fitted to a modified form of Equation 4, using the non-linear least squares modulus in OriginPro:

$$Area = C\left(1 + \frac{k_{th}\, e^{-k_c t} - k_c\, e^{-k_{th} t}}{k_c - k_{th}}\right) + B$$

in which the floating parameters are $k_c$ (rate constant of crystallization), the pre-exponential factor, $C$, and the background, $B$. The threading constants, $k_{th}$, were interpolated at 50 °C and 75 °C from the FTIR Arrhenius plots (Table 1) and fixed in the fits as

| | $k_{th}$ (min$^{-1}$) | |
|---|---|---|
| | 50 °C | 75 °C |
| 1:0.5 | 0.0119 | 0.0370 |
| 1:1 | 0.0108 | 0.0264 |
